# DAF-16 target identification in *C. elegans*: past, present and future

**DOI:** 10.1007/s10522-014-9527-y

**Published:** 2014-08-26

**Authors:** Jennifer M. A. Tullet

**Affiliations:** 1 The Institute of Healthy Ageing, UCL, Gower St., London, WC1E 6BT UK; 2 School of Biosciences, University of Kent, Canterbury, Kent CT2 7NJ UK

**Keywords:** Ageing, *C. elegans*, DAF-16, Insulin/IGF-1 like signalling, mRNA profiling, Proteomics

## Abstract

In *C. elegans*, mutations in the conserved insulin/IGF-1 signaling (IIS) pathway lead to a robust extension in lifespan, improved late life health, and protection from age-related disease. These effects are mediated by the FoxO transcription factor DAF-16 which lies downstream of the IIS kinase cascade. Identifying and functionally testing DAF-16 target genes has been a focal point of ageing research for the last 10 years. Here, I review the recent advances in identifying and understanding IIS/DAF-16 targets. These studies continue to reveal the intricate nature of the IIS/DAF-16 gene regulation network and are helping us to understand the mechanisms that control lifespan. Ageing and age related disease is an area of intense public interest, and the biochemical characterization of the genes involved will be critical for identifying drugs to improve the health of our ageing population.

## Insulin/IGF-1 signaling, DAF-16 and lifespan extension in *C. elegans*

It is possible to dramatically increase the lifespan of an organism by making minute changes to its genetic makeup. In the small nematode worm *Caenorhabditis elegans,* a point mutation in the *daf*-*2* gene (the worm insulin/IGF-1 receptor homologue) is sufficient to double the mean lifespan of the animals by down-regulating the Insulin/IGF-1 signaling (IIS) pathway (Kenyon et al. 1993; Kimura et al. 1997). Mutation of the *age*-*1* gene (the worm phosphatidylinositol 3-kinase homologue which acts downstream of *daf*-*2*) can also increase adult lifespan by up to tenfold (Ayyadevara et al. 2008; Friedman and Johnson 1988). These studies fuelled the ageing field and provided an impetus to investigate how longevity can be regulated by genetic manipulation.

Lifespan extension occurs because *daf*-*2* or *age*-*1* mutation ultimately leads to the de-phosphorylation and nuclear accumulation of the DAF-16 transcription factor (the worm FoxO homologue) (Kenyon et al. 1993; Lin et al. 2001; Ogg et al. 1997). Once in the nucleus, DAF-16 regulates the transcription of a plethora of genes involved in promoting stress resistance, metabolising fat, protecting against pathogens, regulating *C. elegans* dauer formation, and some of which also influence lifespan (Murphy 2006). Other transcription factors also contribute to IIS longevity namely, the HSF-1 (worm Heat Shock Factor), DAF-12 (a worm nuclear receptor), and SKN-1 (the worm Nrf factor) transcription factors (Gems et al. 1998; Hsu et al. 2003; Larsen et al. 1995; Tullet et al. 2008). However, this review will focus on DAF-16 and the genes that it regulates to control ageing.

The finding that the DAF-16 transcription factor is important for lifespan means that biologists can exploit functional genomics analysis to understand how DAF-16 acts. The hope is that by identifying its targets and understanding the biochemical processes they regulate we can discover why *daf*-*2* worms are long lived, understand the ageing process in *C. elegans,* and possibly humans. Despite the vast amount of information gathered on this topic, defining which genes are functionally important for *daf*-*2* lifespan has proved challenging, and it remains one of the big mysteries of biogerontology.

### Using this information to improve late-life health

In addition to simply extending lifespan, there are other good reasons to understand the functions of IIS/DAF-16. Down-regulation of IIS in *C. elegans* strikingly improves the late life health of the animals. Age-related disease states relevant to human ageing can be studied in *C. elegans.* These include Huntington’s and Alzheimer’s disease as well as tumour formation. The protein aggregates associated with Huntington’s or Alzheimer’s are modelled by overexpressing polyglutamine repeats or the Aβ(1-42) peptide, respectively, in specific tissues (Brignull et al. 2006; Cohen et al. 2006; Mohri-Shiomi and Garsin 2008; Morley et al. 2002). Tumour formation can be studied in the *C. elegans* germline where lethal tumours can result from mitotic cells re-entering the cell cycle and hyper proliferating (Pinkston et al. 2006). Reducing IIS via *daf*-*2* or *age*-*1* mutation slows the onset and decreases the severity of all of these pathologies dependent on *daf*-*16* (Cohen et al. 2006; Morley et al. 2002; Pinkston-Gosse and Kenyon 2007).

Like mammals, as they age *C. elegans* exhibit signs of age-dependent decline including a wide range of late life nematode diseases (Gems and de la Guardia 2013). These include: A decline in the integrity of their nervous system characterised by branching of their dendrites and synapse deterioration (Tank et al. 2011; Toth et al. 2012), and a decline in neuronally controlled behaviours as reviewed by (Stein and Murphy 2012); muscle degeneration similar to mammalian sarcopenia, (Garigan et al. 2002; Herndon et al. 2002); a decline in intestinal function characterised by a general loss of gut integrity (McGee et al. 2011); accumulation of yolk (DePina et al. 2011; Herndon et al. 2002); development of large tumours within the uterus (Luo and Murphy 2011; McGee et al. 2011; Riesen et al. 2014); and constipation, which is possibly caused by proliferation of their bacterial food source within the intestinal lumen (Garigan et al. 2002). Although the contribution of individual age-related pathologies in ageing and death is still under investigation, mutation of *daf*-*2* appears to alleviate many of them with the effects typically being dependent on *daf*-*16* (DePina et al. 2011; Garigan et al. 2002; Kauffman et al. 2010; McGee et al. 2011; Tank et al. 2011).

The IIS pathway is evolutionarily conserved and reducing IIS promotes longevity in worms, flies and mice (Fontana et al. 2010). Studies from oldest old humans (including centenarians) have also shown that genetic variants in FOXO3A or the IGF-1 receptor are associated with long life (Anselmi et al. 2009; Flachsbart et al. 2009; Li et al. 2009; Pawlikowska et al. 2009; Soerensen et al. 2010; Suh et al. 2008; Willcox et al. 2008). The pathologies of Alzheimer’s disease are alleviated by reduced IIS in mice suggesting that the link between IIS and age-related disease is also conserved (Cohen et al. 2009). Recent advances demonstrate that lifespan can be increased, and health improved by treating model organisms with drugs that target intracellular signalling pathways (Kennedy and Pennypacker 2013). Together, the conservation of the IIS pathway and its role in regulating age-related decline means that understanding the biochemical nature of its action could help us to manipulate it pharmacologically to improve the late-life health of our population as well as to understand ageing.

## Identification of DAF-16 transcriptional targets

### mRNA profiling

The transcriptional targets of DAF-16, downstream of IIS, have been investigated using microarrays (Budovskaya et al. 2008; Golden and Melov 2004; McElwee et al. 2003, 2004; Murphy et al. 2003; Troemel et al. 2006), serial analysis of gene expression (SAGE) (Halaschek-Wiener et al. 2005), and bioinformatic prediction strategies (Lee et al. 2003). In addition, a recent study from the Murphy lab consolidated a large proportion of this data using a voting algorithm, and identified genes whose expression is consistently altered in response to DAF-16 (Tepper et al. 2013). To give an idea of the numbers, in these consolidated lists 1,663 genes were identified as up-regulated by DAF-16 and 1,733 as down-regulated (Tepper et al. 2013). These studies identified genes with either increased or decreased expression in response to altered IIS, but do not discriminate between direct and indirect targets. The functional annotations of these targets span a wide array of biological processes and have previously been reviewed in detail (Murphy 2006). Genes up-regulated in *daf*-*2* animals include those involved in the stress response and xenobiotic detoxification e.g. glutathione-*S*-transferases (*gsts*), molecular chaperones e.g. heat shock proteins (*hsps*), and antimicrobials e.g. bacterial lysozymes; whilst those down-regulated in *daf*-*2* included genes involved in ubiquitin mediated protein degradation, lipid binding, and ribosomal proteins (McElwee et al. 2004; Murphy et al. 2003; Troemel et al. 2006). In addition, one gene down-regulated by DAF-16 encodes an insulin-like peptide, suggesting that DAF-16 also maintains insulin sensitivity of the animal via positive feedback loops (Murphy et al. 2003). The identification of these genes has led to the conclusion that DAF-16 acts to protect the worms from damage (e.g. heat stress), oxidative stress or pathogens; to promote a favourable metabolic status that allows optimal use of energy resources, and to increase survival in harsh conditions (Halaschek-Wiener et al. 2005; McElwee et al. 2004; Murphy 2006; Murphy et al. 2003).

The role of many of these DAF-16 targets for longevity has been functionally tested; RNAi of many genes up-regulated by *daf*-*2* leads to decreased *daf*-*2* lifespan and RNAi of many genes down-regulated by *daf*-*2* leads to increased wild type lifespan (Murphy 2006). However, no target has entirely recapitulated the effect of the *daf*-*16* mutation itself. This could reflect DAF-16 requiring more than one target gene to control lifespan, or indicate the existence of complex feedback mechanisms where knockdown of one component results in secondary changes (in other genes or molecules) that affect lifespan. In addition, as this analysis has not been exhaustive it is possible that the critical DAF-16 target has not yet been identified.

### Identification of direct DAF-16 target genes

The mRNA profiling experiments described will have identified both direct and indirect DAF-16 target genes. A direct target gene is regulated by DAF-16 at its promoter, with this event leading to a change in gene expression, whereas an indirect target is one whose expression is altered in response to DAF-16 expression, but without direct binding. Two different genome-wide methods have been used to determine the promoters to which DAF-16 binds directly, Chromatin Immunoprecipitation (ChIP) strategies (Niu et al. 2011), and DNA adenine methylation transferase identification (DamID) (Schuster et al. 2010). Using genome wide ChIPSeq, DAF-16 binding events were determined as part of the modENCODE project (Niu et al. 2011). This study used wild-type worms expressing exogenous DAF-16::GFP, but the number of binding events in this study was exceptionally high. This raises concerns that DAF-16 binding might not always correspond to gene regulatory activity, or suggests that the use of overexpressed DAF-16, the GFP tag, or GFP antibody may have increased the risk of identifying unspecific binding events.

Using another approach, Schuster and co-workers used DamID (van Steensel and Henikoff 2000) to monitor where DAF-16 (tagged with a DNA adenine methyl transferase) binds to chromatin by identifying specifically methylated regions of DNA. This is possible because the *C. elegans* genome conveniently has no endogenous DNA methylation. They then cross referenced the DamID data with microarray data (McElwee et al. 2007) which yielded a list of 65 genes whose promoters bound DAF-16, and had altered expression in *daf*-*2* mutants relative to *daf*-*16;daf*-*2* double mutants. Expression of most direct DAF-16 targets overlapped with genes whose expression is increased in *daf*-*2* animals in the microarrays, implying that DAF-16 acts as a transcriptional activator downstream of IIS (Schuster et al. 2010). In contrast to the mRNA profiling data, this list contained only a few genes involved in somatic maintenance (e.g. the stress response genes identified by the mRNA profiling work), but instead was enriched for genes with regulatory functions such as kinases, phosphatases, and transcription factors. Several had already been reported as having effects on ageing. This suggested a model whereby DAF-16 regulates a transcriptional sub-network of other regulators of ageing and thus acts as a regulator of regulators (Schuster et al. 2010). This newer model does not exclude that DAF-16 is regulating genes with direct biochemical effects, indeed as DAF-16 is known to have both cell autonomous and cell non-autonomous effects it is likely that both these regulatory mechanisms are necessary (Libina et al. 2003; Wolkow et al. 2000). However, it may explain the lack of DAF-16 targets able to recapitulate the effects of *daf*-*2,* and introduces a novel testing paradigm.

**Table:** Information box: Summary of techniques used for identifying IIS/DAF-16 mRNA and protein targets

*mRNA profiling* Microarrays and whole genome RNA-Sequencing (RNA-Seq): Used to identify the mRNA signature of a sample or the differences in mRNA expression between samples
*Chromatin profiling* Chromatin Immunoprecipitation (ChIP): Utilizes antibodies specific to the transcription factor of interest (or an associated tag e.g. GFP) to isolate transcription factor-Chromatin complexes. Proteins are then degraded, and any remaining DNA identified either by cloning and sequencing, microarrays, or RNA Seq. DNA Adenine Methyltransferase Identification (DamID): A transcription factor is tagged with a DNA methyl adenosyl transferase that methylates DNA at specific GATC sites within 500 bp of transcription factor binding. Methylated DNA is isolated using the DpnI restriction enzyme and identified using microarrays
*Quantitative Proteomics using Mass Spectrometry* Allows accurate measuring of peptides within a sample by analysing their mass to charge ratio in a Mass Spectrophotometer (MS). Slightly different approaches are used in the studies reported here. In summary: Protein lysates are digested with trypsin and each sample labelled with a different chemically engineered unique mass tag (Depuydt et al. 2013) or isobaric tag (Liang et al. 2014). These samples are then mixed, fractionated, loaded into the MS, and the peptides identified by matching to a database. Samples can be distinguished via their specific tags. Another approach loads each trypsin-digested sample separately on the MS via a High Performance Liquid Chromatography (HPLC) column (Stout et al. 2013). These samples are unlabelled and the identities of peptides are also matched with a database
*Polysome profiling* Also called ribosome profiling. It provides a read out for active mRNA translation: Determining the identity of active ribosomes in a sample and the speed at which they act to control translation. Polysome profiling can also be coupled with mRNA profiling techniques to identify specific mRNA transcripts undergoing translation in different circumstances

### Testing the model that DAF-16 is a regulator of regulators

Recent studies have begun to test the regulator of regulators hypothesis. DAF-16 directly activates the expression of a gamma regulatory subunit of the worm AMP-activated protein kinase (AMPK) (Chen et al. 2013; Tullet et al. 2014). AMPK is a heterotrimeric kinase critical for maintaining energy homeostasis under conditions of low energy (e.g. starvation), and has previously been reported to play a role in ageing (Apfeld et al. 2004; Greer et al. 2007). Notably, DAF-16 is phosphorylated and activated by AMPK suggesting the presence of a positive feedback loop; and recent work supports the existence of such a loop acting downstream of reduced IIS, to increase the rate of DAF-16-dependent transcription, and contribute to *daf*-*2* longevity (Tullet et al. 2014). A similar *daf*-*16*-dependent loop may also occur in a strain of extremely long-lived worms that have both down regulated IIS and S6 Kinase (the kinase downstream of the Target of Rapamycin (TOR) pathway) activity (Chen et al. 2013).

DAF-16 also directly activates transcription of *mdl*-*1*, which encodes the worm Mad basic-loop-helix leucine zipper transcription factor (Riesen et al. 2014). *mdl*-*1* contributes to *daf*-*2* longevity (Murphy et al. 2003; Pinkston-Gosse and Kenyon 2007; Riesen et al. 2014), acting in the intestine and germline where it suppresses hyper-proliferation and uterine tumour formation (Riesen et al. 2014). However, another recent study suggests that DAF-16 down-regulates *mdl*-*1* mRNA levels, indicating that the relationship between IIS and *mdl*-*1* might be more complex, or condition dependent (Johnson et al. 2014).

AMPK and *mdl*-*1* had previously been identified as ageing regulators (Apfeld et al. 2004; Greer et al. 2007; Pinkston-Gosse and Kenyon 2007) but this recent work more precisely defines their place in a DAF-16-centred regulatory network. Their knockdown does not recapitulate the effect of *daf*-*16* on *daf*-*2* lifespan, but their involvement in gene regulatory loops and ageing phenotypes supports a model whereby DAF-16 acts to regulate a number of different regulators. These regulators then act in feedback loops, and each factor makes a small but significant contribution to lifespan.

### Further insights from functional genomics approaches

IIS/DAF-16 regulated mRNA profiling data sets have also been interrogated in various other ways to investigate ageing and lifespan. For example, a detailed bioinformatic analysis integrating DAF-16 mRNA expression profiling and in vivo chromatin binding data of 23 other transcription factors (Niu et al. 2011), queried the fact that while DAF-16 only directly activates gene transcription (via the DAF-16 binding element (DBE)), it also indirectly reduces the expression of many genes (McElwee et al. 2004; Murphy et al. 2003; Schuster et al. 2010). This analysis identified the transcription factor PQM-1 as a regulator of genes previously found to be down-regulated by DAF-16, whose promoters contained a DAF-16-associated element (DAE) (also called a reverse GATA transcription factor binding element) (Tepper et al. 2013). PQM-1 contributes to IIS mediated longevity and is important for the co-ordinated response to stress, development, and lifespan. Future, detailed investigations to further understand its role will be interesting and informative to the field.

In two separate studies the relationship between IIS/DAF-16 targets and disease was addressed. First, *C. elegans* *gld*-*1* mutants develop lethal germline tumours resulting from mitotic cells re-entering the cell cycle and over-proliferating, pathology which is alleviated by mutation of *daf*-*2* (Pinkston et al. 2006). Second, by screening a compilation of DAF-16 targets for their role in tumour formation and identifying overlap with genes that regulate lifespan, new tumour suppressor targets potentially applicable to humans were discovered. Novel, shared mechanisms were identified that control hyper-proliferation leading to tumour formation as well as ageing (Pinkston-Gosse and Kenyon 2007).

Although very different, all these studies demonstrate how IIS/DAF-16 data sets have been useful for: Identifying novel signalling pathways and molecules that regulate ageing, identifying genes important for age related disease, and to question global mechanisms of ageing.

## IIS and DAF-16-dependent changes in the proteome

A natural assumption is that changes in mRNA levels will in turn alter protein levels. However, there is mounting evidence that DAF-16 also exerts a strong post-transcriptional effect. For example, work from the Lithgow lab suggests that DAF-16 could also potentially influence gene expression post transcriptionally to promote heat stress resistance (McColl et al. 2010). Differences in protein expression in *daf*-*2* compared to wild type or *daf*-*16* animals were first analysed using quantitative mass spectrometry by the Yates group (Dong et al. 2007). They identified 86 proteins with altered abundance in *daf*-*2* animals compared to wild-type, a subset of which had previously been identified as DAF-16 transcriptional targets (Dong et al. 2007). More recently the Braeckman and Brenkman groups have used quantitative proteomics to probe the effects of reduced IIS (Depuydt et al. 2013; Stout et al. 2013). The two studies identified 364 and 455 proteins respectively that were changed (increased or decreased) in *daf*-*2* animals compared to wild-type, the identities of which overlapped significantly with those identified by Dong et al. (2007). In each case proteins whose abundance was decreased in *daf*-*2* animals were enriched for functional processes that impact on protein levels (e.g. translation elongation, ribosomal protein subunits, and proteasome components), suggesting that it is a global reduction in protein translation/load that promotes longevity (Depuydt et al. 2013; Dong et al. 2007; Stout et al. 2013) (Table 1). There was also consensus on the functions of proteins identified with increased abundance in *daf*-*2* e.g. redox and carbohydrate metabolism (Table 1). These studies overlapped considerably in terms of the processes identified but less so on an individual gene level (Table 2), although this could be due to technical differences in their strategies, or the fact that one group used one-day old and the other two-day old adult worms.

**Table 1:** Protein functional categories with altered abundance in long lived *C. elegans* mutants

Functional category	Dong et al. (2007) *daf*-*2* vs *daf*-*16; daf*-*2*	Stout et al. (2013) *daf*-*2* vs *daf*-*16; daf*-*2*	Depuydt et al. (2013) *daf*-*2* vs *daf*-*16; daf*-*2*	mRNA profiling
Translation Elongation	Decreased	Decreased		Decreased in old *daf*-*2* (Halaschek-Wiener et al. 2005)
Ribosome		Decreased	Decreased	Increased in old *daf*-*2* (Halaschek-Wiener et al. 2005)
Proteasome core complex		Decreased		Decreased in old *daf*-*2* (Halaschek-Wiener et al. 2005)
Unfolded protein binding			Decreased	
tRNA aminoacylation			Decreased	
Peptidase activity			Decreased	
Gene expression		Decreased		Decreased in old *daf*-*2* (Halaschek-Wiener et al. 2005)
Lipid transport	Decreased			
Amino acid biosynthesis	Increased			
Oxygen and reactive oxygen species metabolism	Increased	Increased	Increased	Increased (Murphy et al. 2003; McElwee et al. 2003, 2004)
Metabolic processes including carbohydrate metabolism	Increased	Increased	Increased	Increased (Murphy et al. 2003; McElwee et al. 2003, 2004) Decreased in old *daf*-*2* (Halaschek-Wiener et al. 2005)
Determination of adult lifespan/ageing		Increased and decreased	Decreased and increased	
Nutrient reservoir activity e.g. vitogellins		Decreased	Decreased	Decreased (Murphy et al. 2003; Halaschek-Wiener et al. 2005)
Growth, reproduction and developmental processes		Decreased	Decreased (molting/cuticle) and increased (positive regulators of growth e.g. muscle proteins)	

Three proteomic studies comparing *daf*-*2* vs *daf*-*16; daf*-*2* animals are reported (Depuydt et al. 2013; Dong et al. 2007; Stout et al. 2013). In some cases functional and GO categories have been combined to give a more general overview of the processes affected

**Table 2:** Individual genes whose protein products are altered in *daf*-*2* vs *daf*-*16; daf*-*2* animals

Gene	Protein name	Protein function
*Proteins with increased abundance in daf-2(e1370)*		
*alh*-9*	Aldehyde dehydrogenase	Predicted to catalyse aldehyde oxidation; *alh*-*9(RNAi)* results in embryonic and larval lethality, slow growth, sterility and body morphology defects
B0286.3	No info	
C31C9.2	No info	
*cdr*-*2*	Cadmium responsive	
*dpy*-*11*	Dumpy: shorter than WT	Thioredoxin- like protein that affects body shape and ray morphology
F09B12.3	No info	
F37C4.5	No info	
*fbp*-1*	Fructose-1,6-biphosphatase	Gluconeogenic enzyme that catalyses the hydrolysis of fructose 1,6-bisphosphate to fructose 6-phosphate and inorganic phosphate in a reaction that reverses the third enzymatic step of glycolysis
*gei*-*15*	GEX Interacting protein	
*gei*-*7* */icl*-1*	Isocitrate lyase/malate synthase homolog	Predicted to function in the glyoxylate cycle; ICL-1 is required for embryonic morphogenesis and appears to act downstream of DAF-16 to influence lifespan
*glb*-1*	Globin related	Repressed by DAF-2 signalling in a DAF-16 dependent manner
*gst*-*1*	Glutathione *S*-transferase	Required for sperm migration
*gst*-10*	Glutathione *S*-transferase	Catalyses the conjugation of glutathione to 4-hydroxynonenal in vitro
*isp*-*1*	Iron-sulfur protein	
*lbp*-*6*	Lipid binding protein	
*lea*-1*	Plant late embryo abundant related	
*lec*-*4*	Galectin	Exhibits sugar binding properties in vitro
*lec*-*5*	Galectin	
*lec*-6*	Galectin	LEC-6 can interact with several types of blood group precursor oligosaccharides and gangliosides in vitro
*mdt*-*28*	Mediator	Transcriptional regulatory complex
*myo*-*3*	Myosin heavy chain (MHC) minor isoform	Essential for thick filament formation, for viability, movement, and embryonic elongation
*npa*-1*	Nematode polyprotein allergen related	Encodes a large polyprotein precursor that is post-translationally cleaved to multiple units of ~14.5 kDa, each of which is a strong binding protein for fatty acids and retinol; NPA-1-derived peptides are probably carrier proteins that enable lipid distribution in nematodes; NPA-1-derived peptides are also secreted by parasitic nematode species
*pgk*-*1*	Phosphoglycerate kinase	
*spc*-*1*	Spectrin	Required for body morphogenesis, formation of body wall muscles, locomotion, and larval development
T25B9.9	No info	
W10C8.5*	No info	
*Proteins with decreased abundance in * *daf*-*2(e1370)*		
C44B12.1	Permeable eggshell	
*cgh*-1**	Conserved germline helicase	Inhibits physiological apoptosis in oocytes; required for sperm function, oocyte fertilization, and early embryonic cytokinesis
D2096.8*	Nucleosome assembly protein	Required for transcriptional regulation that affects a number of biological processes including embryonic and larval development; Previously noted to be down in *daf*-*2* animals compared to WT
*daf*-21*	Abnormal dauer formation (Hsp90 family)	Molecular chaperone; required for larval development, negative regulation of dauer formation, and a number of specific chemosensory behaviours; required for the extended life span seen in *age*-*1* mutant animals
*eft*-2*	Eukaryotic translation elongation factor 2	GTP-binding protein essential for the elongation phase of protein synthesis; *eef*-*2* is required for embryogenesis and vulval morphogenesis
F13H8.7	Ureidopropionase beta	Catalyses the catabolism of 3-ureidopropionate and 2-methyl-3-ureidopropionate
*hsp*-*1*	Heat shock protein *(hsp70A)*	*hsp*-*1(RNAi)* results in a small reduction of *age*-*1* life-span
*hsp*-*6*	Heat shock protein	Mitochondrion-specific chaperone; involved in the mitochondrial unfolded protein response; required for normal growth and development; HSP-6 levels are markedly reduced in aged worms
*imb*-*3*	Importin beta family	Predicted to function as a nuclear transport factor that, with the RAN-1 GTPase, regulates nuclear import of ribosomal proteins; IMB-3 is essential for embryogenesis and germline development, and may also be required for normal postembryonic growth rates
K07C5.4	No info	
*lys*-*1*	Lysozyme	Pathogen resistance
M28.5	No info	
*nasp*-*2*	NASP (human nuclear autoantigenic sperm protein) homolog	
*pas*-*6*	Proteasome type 1 alpha subunit of 26S 20S core particle	PAS-6 is required for embryonic, larval, and germline development
R07H5.8	No info	
R09B3.3	No info	
*rack*-*1*	RACK1 (mammalian receptor of activated C kinase) homolog	Required cell autonomously for VD/DD motor axon pathfinding; *rack*-*1* is also required for gonadal distal tip cell migration and for normal brood sizes
*rpa*-*0/rla*-*0*	Ribosomal protein, large subunit, Acidic (P1)	
*rpl*-*19*	Ribosomal protein, large subunit	
*rpl*-*25.2*	Ribosomal protein, large subunit	
*rpl*-*31*	Ribosomal protein, large subunit	
*rpl*-*4*	Ribosomal protein, large subunit	
*rps*-*15*	Ribosomal protein, small subunit	
*rps*-*16*	Ribosomal protein, small subunit	
*rps*-*24*	Ribosomal protein, small subunit	RPS-24 activity is required for germline development and the overall health of the animal
*sams*-*1*	*S*-adenosyl methionine synthetase	*sams*-*1(RNAi)* can extend adult lifespan
*vit*-1*	Vitellogenin structural genes (yolk protein genes)	
*vit*-3*	Vitellogenin structural genes (yolk protein genes)	VIT-3 is a major yolk component but *vit*-*3(RNAi)* does not result in any abnormalities, VIT-3 likely functions redundantly with other vitellogenins to provide essential nutrients to the developing embryo
*vit*-5*	Vitellogenin structural genes (yolk protein genes)	Predicted to function as a lipid transport protein; *vit*-*5(RNAi)* indicates that VIT-5 is required for embryogenesis and normal rates of postembryonic growth
*vit*-6*	Vitellogenin structural genes (yolk protein genes)	Vitellogenin precursor protein that is cleaved in the body cavity into two smaller yolk proteins, YP115 and YP88

These proteins were identified as altered by both (Depuydt et al. 2013; Stout et al. 2013). Those in red are also less abundant in the proteome of aged (5 or 10 day old adults) compared to young (1 day adult) worms; those in blue are more abundant in aged worms (Liang et al. 2014)

* This protein was also altered in the same direction in (Dong et al. 2007)

** RNAi knockdown of this gene extended lifespan of N2 worms but not *daf*-*16(mu86)* mutants (Stout et al. 2013)

### Decreased translation as a potential mechanism for* daf-2 *longevity

Polysome profiling is the quantification of ribosomes contained on actively translated mRNA molecules. A decreased number of polysomes were observed in *daf*-*2* animals compared to wild type, and this was *daf*-*16*-dependent suggesting that IIS can specifically control polysomal mRNA translation (Stout et al. 2013). *daf*-*2* mutants also displayed repressed levels of protein synthesis using ^35^S methionine incorporation (Depuydt et al. 2013), although this was not seen in a previous study (Hansen et al. 2007). Decreasing translation can extend *C. elegans* lifespan. Mutation of *ife*-*2* or RNAi knockdown of *ifg*-*1* (worm eIF4E and eIF4G translation initiation factors); RNAi of *eef*-*2*, *eef*-*1A.2* or *eef*-*1B.1* (worm translation elongation factors); RNAi of *rps* and *rpl* genes (worm small and large ribosomal subunits); or mutation of *rsks*-*1* (the worm S6 Kinase homologue which acts downstream of TOR) all extend lifespan compared to wild type controls (Hansen et al. 2007; Li et al. 2011; Pan et al. 2007; Wang et al. 2010). Decreased translation may also mediate the increased lifespan observed in response to dietary restriction (DR) or mutations in the TOR pathway (Hansen et al. 2007; Kapahi et al. 2010; Stanfel et al. 2009), but was not previously thought to contribute to *daf*-*2* longevity. Interestingly, the *daf*-*2* proteomics data significantly resembles those of DR worms, particularly with respect to proteins with translational function (Depuydt et al. 2013), suggesting a shared mechanism of action, and the new polysome profiling data also supports a model where translation is important for *daf*-*2* lifespan (Stout et al. 2013). *daf*-*2* mutants differ from wild type animals in many respects other than increased longevity, i.e. they are dauer constitutive and exhibit delayed development. Thus, it may be that reduced translation does play a role in *daf*-*2* longevity, but that other processes contribute as well. DR or TOR mutants could be considered less complex, with translation being a more substantial contributing factor to their longevity.

Previously, the mechanisms responsible for the longevity of IIS mutants were thought to be different from those underlying the longevity of TOR mutants or animals subjected to DR. Mutation of *let*-*363* (the worm TOR orthologue) together with *daf*-*2* does not have an additive effect on lifespan (Vellai et al. 2003) suggesting that the two pathways act by common mechanisms. In contrast, mutation of *rsks*-*1* together with *daf*-*2* results in a strain that far outlives either of the single mutations (Chen et al. 2013) suggesting non-overlapping mechanisms of action. As the *daf*-*2* animals used in these experiments are not completely null, accurate interpretation of these epistasis experiments is difficult. It is likely that several different biological process will coordinate to regulate lifespan, and that different processes will be pertinent in specific situations. However, if shared longevity mechanisms between IIS and TOR do exist then one candidate process might be autophagy (Hansen et al. 2008, 2007; Melendez et al. 2003).

### Other mechanisms controlling* daf-2* longevity suggested by proteomic studies

The *daf*-*16*-dependent reduction in protein translation observed in *daf*-*2* mutants is accompanied by a 48 % reduction in total mRNA and a decrease in proteasome activity (Stout et al. 2013). Thus, an alternative mechanism supported by the proteomics data is that changes in the synthesis and breakdown of proteins (protein metabolism) may affect lifespan.

As worms age they exhibit decreased muscle integrity similar to mammalian sarcopenia, but *daf*-*2* mutation protects against this (Garigan et al. 2002; Herndon et al. 2002). In contrast, both *daf*-*2* mutants and DR animals have an increased abundance of muscle related proteins (Depuydt et al. 2013; Stout et al. 2013) which correlates with an increase in the preservation of their muscle volume relative to a decrease in their whole body volume (Depuydt et al. 2013). This can be attributed to an DAF-16 driven up-regulation of mRNAs encoding muscle genes but the authors suggest that complementary mechanisms, e.g. tissue or target specific decreased protein degradation or suppression of protein synthesis, could also contribute (Depuydt et al. 2013). This data suggests a positive role for maintenance of muscle integrity in promoting longevity and poses an important area of research, highly applicable to human sarcopenia.

### Relating the* daf-16*-dependent transcriptome and proteome

What is striking about the studies showing *daf*-*2* induced changes in the proteome is that they are not mirrored at the mRNA level despite being dependent on the transcription factor DAF-16, which presumably regulates gene transcription, and therefore mRNA levels (Table 1) (Depuydt et al. 2013; Stout et al. 2013). In this case, to link the process of transcription with the proteome, one needs to identify an intermediate gene whose transcription is regulated by *daf*-*16*, and whose activity impacts on the proteome as with a post-transcriptional effector. One candidate, suggested by the Brenkman group, is *S*-adenosyl methionine synthase-1 (*sams*-*1*) that has decreased mRNA levels and protein abundance in *daf*-*2* animals (Depuydt et al. 2013; Dong et al. 2007; McElwee et al. 2004; Stout et al. 2013). These differences are *daf*-*16*-dependent but may not be direct (Oh et al. 2006; Schuster et al. 2010). SAMS-1 is responsible for the universal methyl group donor (*S*-adenosylmethionine), and knock down of *sams*-*1* has been reported to dramatically decrease global protein translation (Ching et al. 2010), so that might explain the decreased translation observed in *daf*-*2* animals. However, *sams*-*1* RNAi can extend the lifespan of *daf*-*2* mutants suggesting that either *sams*-*1* mediated processes cannot be the only mechanism required for IIS longevity, or that they converge on the same mechanisms and result in an additive effect (Gems et al. 2002; Hansen et al. 2005).

**Fig. 1:**
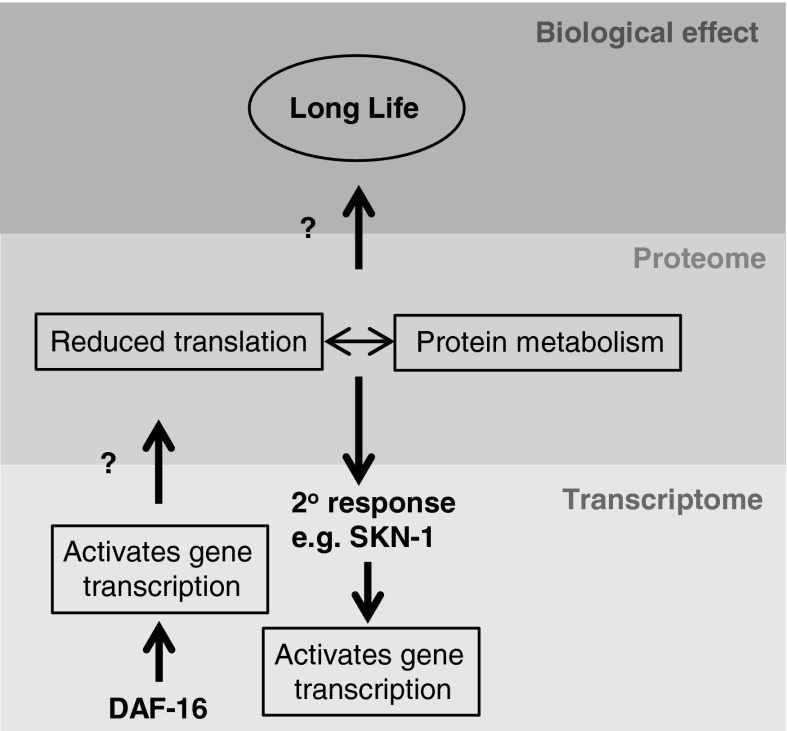
Relationship between the *C. elegans* transcriptome and proteome. Adapted from the conclusions drawn by Stout et al. (2013). In response to reduced IIS DAF-16 binds to chromatin and directly activates gene transcription and alters the proteome. These changes lead to altered protein metabolism coupled with a decrease in translation. Reduced translation is stressful and activates a secondary SKN-1-dependent transcriptional response (Robida-Stubbs et al. 2012) making it difficult to separate translation and transcription. The mechanisms through which reduced translation and altered protein metabolism promote longevity are under investigation

Reducing protein synthesis can be stressful to the organism (Hansen et al. 2007; Pan et al. 2007; Robida-Stubbs et al. 2012). This can be indicated by the activation of the stress response transcription factor SKN-1. For instance, RNAi knockdown of the TORC1 specific gene *ragc*-*1* decreases protein synthesis and activates a SKN-1-dependent protective transcriptional response (Robida-Stubbs et al. 2012), as does inhibiting translation initiation (Wang et al. 2010). SKN-1 is required for the longevity incurred by reduced TORC1 signalling and reduced translation initiation (Robida-Stubbs et al. 2012; Wang et al. 2010). It also promotes longevity downstream of *daf*-*2* and is regulated by the AKT and SGK kinases in parallel to DAF-16 (Kenyon 2010; Tullet et al. 2008). Thus, if IIS and DAF-16 alter the proteome, this will impact on the activity of SKN-1, incur secondary transcriptional responses, and make it difficult to separate transcriptional and translational control of longevity (Fig. 1).

These proteomics studies are intriguing in several respects. First, they suggest novel mechanism(s) by which reduced IIS promotes longevity. As wild-type worms age they accumulate yolk proteins and protein aggregates, but *daf*-*2* mutation protects against this (David et al. 2010; DePina et al. 2011; Depuydt et al. 2013; Liang et al. 2014; Reis-Rodrigues et al. 2012; Stout et al. 2013). Mechanisms that decrease protein load in *daf*-*2* animals could explain these effects. Second, due to their discrepancies with the mRNA profiling data they also raise questions about using mRNA profiling strategies to uncover mechanisms of ageing. In regard to this second point, it is worth noting that whilst in mammalian cell culture (in vitro), the correlation between the transcriptome and proteome is high, in individual human tissues (in vivo) the correlation between these data sets is not as good (Wilhelm et al. 2014). In the study using human tissues, the authors put these discrepancies down to the mixture of cell types in each tissue. However, comparing human mRNA profiling and proteomic data shows that whilst the actual amount of protein in each tissue differs, the translation rate is constant, and so the actual amount of protein in a given cell type is determined by regulating the mRNA levels (Wilhelm et al. 2014). As the majority of all worm profiling data uses whole worms, similar reasoning could be applied to these studies.

Ultimately, the relevance and usefulness of any genome wide approach lies with the functional testing of the candidates that it identifies. In terms of testing candidate genes and mechanisms the mRNA profiling studies are by far the most advanced. The proteomic studies begin to functionally test their candidates, but so far none have recapitulated the dramatic lifespan effects seen in *daf*-*2* animals (Stout et al. 2013). It is too early to say whether one approach yields a higher success rate than the other in terms of understanding ageing but it is worth considering that the proteome is possibly more physiologically relevant than the transcriptome, and studying it should provide valuable information on this complex process. Overall, both the study of genes and proteins will contribute to the increased understanding of the mechanisms of ageing.

## Future perspectives

Our understanding of how IIS regulates ageing and lifespan through DAF-16 is progressing: The whole-genome studies that I have reviewed here increase our knowledge of how DAF-16 interacts with specific target genes, identify novel potential mechanisms for IIS action, and suggest new models to explain ageing. However, a complete model of exactly how DAF-16 responds to reduced IIS to slow ageing is still not clear.

In the profiling experiments reviewed here, *daf*-*2* mutants are nearly always compared to *daf*-*16;daf*-*2* double mutants in order to identify DAF-16 targets that are responsible for mediating the extreme longevity of *daf*-*2* animals. It is hoped that in doing this we will also identify genes required for normal ageing, longevity, and health. However, whilst *daf*-*16* is necessary for lifespan, it is not sufficient under normal circumstances, and increasing DAF-16 expression using transgenic arrays does not lead to increased lifespan (Henderson and Johnson 2001). Therefore, these comparisons are useful, but may not identify all factors required for longevity. More recently, it has been shown that treating worms with 5′Fluorodeoxyuridine reveals an increased lifespan in animals overexpressing DAF-16 by inhibiting over-proliferation of germline cells (Alic et al. 2014; Qi et al. 2012). Although this may not represent a perfect model for DAF-16 action it does provide a model with which to examine DAF-16 mediated longevity in more detail. Future work to examine the targets of DAF-16 in these models will be very interesting.

A shortcoming of all the reported IIS profiling experiments is that they use whole worm extracts. However, DAF-16 is known to regulate its targets in both cell autonomous and cell non-autonomous ways (Alic et al. 2014; Zhang et al. 2013), so it could be argued that using the whole organism gives the best overall picture. However, this will inevitably lead to an under-representation of genes from smaller tissues. New techniques allowing tissue specificity either at the level of mRNA isolation (Von Stetina et al. 2007) or bioinformatically post-array (Chikina et al. 2009), could in future improve our understanding of DAF-16 action. Single cell RNA-Seq will also provide a powerful technique for mRNA profiling studies (Hashimshony et al. 2012). Another issue is that gene regulatory function changes depending on genetic background and the environment, so it is difficult to make studies fully comprehensive. Specific biological questions must therefore be addressed with carefully designed functional genomics experiments.

For a long time the major focus to understanding DAF-16 function has been directed at its transcriptional targets, however this recent increase in the number of proteomics papers is striking. There is no doubt that the mRNA profiling work has, and continues to, provide us with a great deal of knowledge on how DAF-16 is working. The proteomic studies on the other hand, demonstrate that biologists are broadening their approach to elucidate new models of IIS/DAF-16 action that also challenge the way we think about ageing itself. So what should the conscientious scientist do? It is almost certain that there is more to discover by individually examining DAF-16 targets identified by mRNA profiling. Similarly, individual follow up on novel targets identified by proteomics will be valuable. So, rather than dropping one technique in favour of another, it is likely that a combination of both approaches will be valuable in understanding DAF-16 action and ageing. Indeed, defining how the transcriptome and proteome interact, and their influence on each other, will be fascinating to interpret whilst being critically important to understanding the biology of an organism as a whole. In general the experimental technique should reflect the scientific question, but these new studies certainly highlight the usefulness of proteomics, and future studies aimed at understanding the function of other IIS regulated transcription factors (e.g. SKN-1 and HSF-1) will also benefit from this approach. 

There is still more work to be done investigating individual DAF-16 target genes identified from mRNA and chromatin profiling work. The model in which DAF-16 is acting as a regulator of regulators looks promising, and there are more interesting, DAF-16 regulated, candidates (e.g. *skn*-*1*) to follow up on. Work has also started on following up on individual IIS/DAF-16 targets identified by proteomics. However, if reduction of an individual mRNA does not necessarily correspond a reduction in its translated protein (possibly due to issues with protein turnover) then RNAi might not be the best way to functionally test these genes. Genetic nulls or Clustered Regularly Interspaced Short Palindromic Repeats—Cas gene technology (CRISPR-Cas9) for gene editing (Frokjaer-Jensen 2013) provide cleaner, more absolute, models for gene knockdown and can be utilized where appropriate. However, an issue with both genetic nulls or CRISPR generated mutants is that they have the potential to subject the animal to developmental changes that are unrelated to ageing. RNAi greatly enhances the information we gain regarding the temporal control of the gene. Thus, a combination of both genetic mutants and RNAi are the *C. elegans* geneticist’s strongest weapon, whilst a technology that completely knocks down gene function in a conditional manner would be ideal. In addition, chemical strategies that block translation or proteasome function will also be informative.

It is important to harness this information and link it to age-related disease. IIS and DAF-16 are key players in numerous pathologies and diseases associated with age, and *C. elegans* models of these disease states combined with the strength of the worm as a genetic model will facilitate rapid screening of DAF-16 target genes for their roles in age-related disease. This will allow us to identify DAF-16 targets that globally protect late-life health in a similar way to *daf*-*2* mutation, and/or to determine targets or families of targets that are important for specific disease states. This knowledge can then be harnessed pharmacologically to improve the late-life health of our ageing population.
